# Supplementary material for: Views and Perceptions of People Aged 55+ on the Vaccination Programme for Older Adults in the UK: A Qualitative Study
Source: Vaccines (Basel). 2023 Apr 20;11(4):870. doi: 10.3390/vaccines11040870 (PMC10144107; doi:10.3390/vaccines11040870)
Supplement: Supplementary file 1 [file vaccines-11-00870-s001.zip › vaccines-2291376-supplementary.pdf]

Table S1. Themes and subcategories

**1. Personal aspects**			
	1.1 Decision-making		
		A. Perceptions of benefits and risks	
			A1. Perceived benefit
			A2. Perceived risk
		B. Unquestioning support	
			B1. Lack of agency
			B2. Habitual behaviour
		C. Personal choice – non-vaccination	
		D. Scientific change - barriers	
			D1. Vaccine technology
	1.2 Previous experiences		
		A. Young age	
			A1. School
			A2. Upbringing
		B. Family history	
		C. Travel	
		D. Employment related	
		E. Acting as carer	
			E1. Uncertainty when caring for others
**2. Interpersonal aspects**			
	2.1 Friends and family as sources of information		
		A. Social influences	
			A1. Influencing others

**3. Wider community and cultural aspects**			
	3.1 Hesitance among others		
		A. Lack of understanding of opposing views	
		B. Respecting personal choice	
		C. Non-vaccination in care sector	
	3.2 Alternatives to vaccination		
		A. Precautions – alternatives to vaccination	
			A1. Positive or proactive precautions
			A2. Negative or limiting precautions
		B. Natural immunity	
**4. System related aspects**			
	4.1 Access		
		A. Access barriers	
			A1. GP access in general
			A2. Access to other vaccines
		B. Ease of access	
			B1. Pharmacies
		C. Lack of choice	
	4.2 Information		
		A. Lack of information	
			A1. GP
			A2. Awareness, ambivalent
			A3. Unreliable information
			A4. Misinformation

	B. Healthcare professionals	
		B1. Suggestions for uptake
	C. Media – barriers and concerns	
		C1. Social media
	D. Science and experts	
		D1. Importance of research
	E. Non-mainstream research	
4.3 Policy		
	A. National aspects	
	B. Alternatives to vaccinating	
		B1. Older adults
		B2. Prioritising young age groups
		B3. Risk based priority groups
	C. Mistrust	
		C1. Pharmaceuticals

Supplementary S1

Focus Group Topic Guide

<u>Introductions</u>

- Brief introduction to the discussion, reminding participants of the purpose of the research. Preamble from participant information sheet (Supplementary S2) will be discussed, reminding participants of the NHS vaccination offer for the 65+ age group and the 3 vaccinations that form the basis of the conversation. Reminding that COVID-19 vaccination will be discussed towards the end of the focus group.
- Reminder about consent and giving the option to opt out of the video call if not feeling comfortable with being recorded.
- Explanation of how the session will run. This includes reminding participants to be respectful of others, to wait for their turn to speak using the raise hand function to facilitate this and overview of using the chat function, as well as using the mute and video off function if they would like to take a comfort break. Reminder that if

participants feel distressed at any point, they can let the facilitator know via the private chat function, turn off their video/audio and one of the facilitators will contact them to advise of support available.

- Details of support services for bereavement, mental health support and health advice will be shared with any distressed participants. These are the National Bereavement Partnership telephone helpline; nation-specific mental health support telephone lines; Crisis text line phone number and NHS website and phone number. Details for Hub of Hope will also be shared for identifying local support services.

- Start discussion by inviting the participants to introduce themselves by saying a few words if they would like to. This is also to check for any difficulties using Zoom.

General

What do you think about the vaccines available to people aged 65 and over?

- Are you aware of the vaccinations available to 65+ age group free of charge?
- Did you/would you get vaccinated when you are invited to do so?
- Are some vaccines more important than others to you?
- What makes a vaccine relevant to you?

Do you feel it is easy for you to get vaccinated if you wanted to?

- What might make getting vaccinated easier?
- Is there something you would like to change about how you get vaccinated?

Beliefs

- How might your personal or cultural views and beliefs relate to getting vaccinated? Do you have any expectations about vaccines or vaccination?
- What made you feel that way?
- How did you feel last time when you were getting vaccinated? What about after the vaccination?

Is there anything about getting vaccinated that you find unpleasant or that causes distress to you?

- Are there any habits or customs that might encourage or stop you from being vaccinated in general? (probing for religion/culture)

Communication

What kind of things help you decide whether you want to be vaccinated?

- Is there something that might make you change your opinion about vaccination?
- How are you informed about vaccines?

Is there someone that might help you make a decision to be vaccinated (or not)?

- Do you receive information from your GP practice about vaccination?

What about the influence of family and/or friends?

- Have you recommended a vaccine to someone based on your own experiences?
- Would you listen to advice given by a family member or friend?
- How might an experience a family member or friend has had about vaccination affect your decision making?

What about the information available via different media outlets?

- How might media affect your likelihood of getting vaccinated (TV, radio, newspapers, social media)?
- Where do you find information about vaccines?
- Do you feel you have enough information about the vaccines available to you?

<u>Alternatives</u>

Apart from getting vaccinated yourself, what else might provide you with protection against infectious disease?

- Do you see there being any alternatives to vaccination?
- How important do you think it is to vaccinate older adults specifically? How do you feel about others being vaccinated to offer protection to you (herd effects/protection, for example flu)
- Might there be any alternatives to this?

<u>Current Circumstances</u>

Has the COVID-19 vaccination programme affected your opinion on vaccines in general?

- How does your social life affect your need to be vaccinated?

<u>Specific Vaccines</u>

What might make you think a certain vaccine would be more important to you?

Why might you choose to get a certain vaccine?

Close

Is there anything else you would like to talk about in terms of vaccination?

Thank participants and share information about compensation.

Supplementary S2

Supplementary S2 includes additional quotes from participants that have been excluded from the main text to limit the length of the main text.

Quote 1:

> "I have a real issue with people sticking needles into me. I know it's all in the head but no matter how hard I try and rationalise it… so I always warn people doing a vaccine, as I did with the various COVID ones and flu's I've had recently, that if I turn white, go sweaty, and look faint, that's a perfectly normal reaction for me. And I've found over the years—because the first time I didn't tell someone, they thought something really horrible was happening. I just said I have this reaction to people sticking needles in me. I can't explain it, I don't want to have it, but it happens" (G3, P4, age 71).

Quote 2:

"Oh yes, of course, but on an individual basis as each vaccine either was being produced or I felt it was needed or someone was saying, I needed it, I would evaluate it on an individual basis" (G13, P3, age 79).

Quote 3:

"For me, there was no question that you got inoculated, and I've travelled quite a lot since then so, whenever you went anywhere, it's standard practice—go to the surgery, see what jabs you need, see whether you've had them, 'Are they in date?' I actually had a whole load of vaccinations in March 2020 because we were meant to be going to Australia in that September but, sadly, we didn't…" (G5, P2, age 57).

Quote 4:

"The one discussion I can think of in my family was my mother who died almost a year ago, and she didn't want to have the vaccine, not specifically because any dislike of having vaccines, but she knew that she was gonna be dying soon, and she didn't want to use up a vaccine that could go to someone else… In terms of friends—I cannot think of any friends I've got who have been anything other than very clearly—and quite proactively at times—enthusiastic about vaccines and encouraging people to get them." (G5, P3, age 55).

Quote 5:

"...There was another friend and… she wouldn't have the [COVID-19] vaccine even though her son was actually in a vulnerable category and was having the vaccine early as a result of that. And I found that so hard and she's one of my oldest, oldest friends. You know we've known each other since we were five years old and I avoided talking to her about why she didn't want it because I just felt—I wasn't gonna change her mind right so I wasn't gonna save her and all it was gonna do was lead to some sort of conflict"(G9, P1, age 56).

Quote 6:

"I worry that the next generation won't have that same experiences and they won't have their grandparents there telling them that they knew people who had suffered and I suspect that the uptake of vaccinations may well decline in the future" (G10, P4, age 63).

Quote 7:

"I think because she comes from, originally from Bangladesh and I think the country who value vaccination very highly in the way of keeping children safe but she's emphatic that she's not having any vaccination" (G9, P2, age 69).

Quote 8:

"I'm just thinking about it now reflecting on how I managed to get whooping cough. I'm thinking perhaps I didn't have it [the vaccine] because my mum would have sort of thought if I am going to get something God will make sure that I get it or I don't get it, you know that kind of thinking and I'm so far away from that sort of thinking but I do believe a lot of people tend to think like that" (G10, P2, age 66).

Quote 9:

"Not only do people endanger themselves by not really believing these things, but they endanger me as a person, as well and restrict my movement by them being in my company. That's what they do and that's what my angst is about people, in general about that situation" (G11, P1, age 69).

Quote 10:

"…last weekend, I went out on a social event with people I didn't know in a crowded venue for the first time, partly as a favour to someone else—partly to go with them. I probably wouldn't have gone on my own but—I've got to admit—when I came back, I did use a mouthwash—did a bit of gargling, 'cause it costs nothing, and there is some research to say it's helpful. I'm continuing to wear a mask in crowded places…"(G6, P1, age 62)

Quote 11:

"I think one of the things is that people should be prepared before they reach 65 to say that these are vaccinations that maybe will be coming down the line to you rather than it suddenly hitting you when you hit 65 and then all of these things sort of happen so there doesn't seem to be an awful lot of pre information that prepares you for what you might have available or what you might want to enquire about" (G10, P5, age 77).
